# Comprehensive comparison of local effect model IV predictions with the particle irradiation data ensemble

**DOI:** 10.1002/mp.15343

**Published:** 2021-12-01

**Authors:** Tabea Pfuhl, Thomas Friedrich, Michael Scholz

**Affiliations:** ^1^ Biophysics Department GSI Helmholtzzentrum für Schwerionenforschung Darmstadt Germany; ^2^ Institute for Solid State Physics Technische Universität Darmstadt Darmstadt Germany

**Keywords:** local effect model, model validation, PIDE, RBE

## Abstract

**Purpose:**

The increased relative biological effectiveness (RBE) of ions is one of the key benefits of ion radiotherapy compared to conventional radiotherapy with photons. To account for the increased RBE of ions during the process of ion radiotherapy treatment planning, a robust model for RBE predictions is indispensable. Currently, at several ion therapy centers the local effect model I (LEM I) is applied to predict the RBE, which varies with biological and physical impacting factors. After the introduction of LEM I, several model improvements were implemented, leading to the current version, LEM IV, which is systematically tested in this study.

**Methods:**

As a comprehensive RBE model should give consistent results for a large variety of ion species and energies, the particle irradiation data ensemble (PIDE) is used to systematically validate the LEM IV. The database covers over 1100 photon and ion survival experiments in form of their linear‐quadratic parameters for a wide range of ion types and energies. This makes the database an optimal tool to challenge the systematic dependencies of the RBE model. After appropriate filtering of the database, 571 experiments were identified and used as test data.

**Results:**

The study confirms that the LEM IV reflects the RBE systematics observed in measurements well. It is able to reproduce the dependence of RBE on the linear energy transfer (LET) as well as on the α_γ_/β_γ_ ratio for several ion species in a wide energy range. Additionally, the systematic quantitative analysis revealed precision capabilities and limits of the model. At lower LET values, the LEM IV tends to underestimate the RBE with an increasing underestimation with increasing atomic number of the ion. At higher LET values, the LEM IV overestimates the RBE for protons or helium ions, whereas the predictions for heavier ions match experimental data well.

**Conclusions:**

The LEM IV is able to predict general RBE characteristics for several ion species in a broad energy range. The accuracy of the predictions is reasonable considering the small number of input parameters needed by the model. The detailed quantification of possible systematic deviations, however, enables to identify not only strengths but also limitations of the model. The gained knowledge can be used to develop model adjustments to further improve the model accuracy, which is on the way.

## 1 INTRODUCTION

The increased relative biological effectiveness (RBE) of ions is one of the key benefits concerning their application in cancer radiation therapy in comparison to conventional radiotherapy with photons. As in a typical irradiation scenario a mixture of different ion species and energies is present in the irradiation field, the effectiveness of each radiation species needs to be predicted with an RBE model in order to perform accurate dose optimizations. Thus, the continuous improvement of clinically used RBE models is a key factor for the further improvement of ion therapy treatment for cancer patients. To safely apply a model, its strengths and limitations have to be carefully determined such that potential countermeasures can be developed. An extensive set of measurement data is optimal to benchmark a model since systematic model dependencies are recognized best if the model is validated against a big dataset. By model comparisons to single measurements, such dependencies potentially stay hidden. In this work, RBE predictions of the local effect model (LEM)^1^, ^2^, ^3^, ^4^ are comprehensively compared to RBE values that are calculated from measured linear–quadratic (LQ) parameters provided in the PIDE database.^5^, ^6^ The investigations are based on the current version of the RBE model, LEM IV.^3^, ^4^ First model tests of the LEM IV based on the PIDE were conducted for carbon ions in Friedrich et al.^5^ with a previous version of the PIDE (PIDE 2.0). In the publication, the accuracy of RBE predictions as a function of β_γ_/α_γ_ and β_i_ as a function of linear energy transfer (LET) is shown for a subset of PIDE data on carbon ions. The PIDE was extended since then from 855 experiments (extracted from 77 publications) to 1118 experiments (extracted from 115 publications). Furthermore, the presented study investigates several other ion species, other predicted quantities, associated error bars, and other dependencies of interest to assess the accuracy of an RBE model. The LEM IV has shown good agreement with measurement data in several previous model applications.^3^, ^4^, ^7^, ^8^, ^9^, ^10^, ^11^ However, single comparisons of LEM IV predictions to measurement data already revealed a tendency toward an underestimation of RBE at higher ion energies for carbon ions. This was observed, for example, for V79 Chinese hamster cells in vitro^4^ as well as for the tolerance of rat spinal cord in vivo.^12^ Furthermore, the same trend was found in an in vitro study with four different tumor cell lines.^13^ Therefore, an important aim of the present study was to further characterize and quantify this potentially systematic trend. Although the quantification of the model improvement with a single number (e.g., *χ* ^2^) seems desirable, it would not be very meaningful as the model precision depends on many factors as LET range, cell line, or ion species. Comparisons of LEM IV to LEM I are published in previous studies.^12^, ^14^, ^15^

The systematic testing of the LEM IV was performed by comparing model predictions to cell survival measurement data of the PIDE database. The database was introduced in 2013 and updated several times since.^5^, ^6^ It is freely available under www.gsi.de/bio‐pide. Since 2019 an update is available (PIDE 3.2) including the raw data of each cell survival experiment, provided that the survival curves are given in the original publication.^6^ If the raw data are available, LQ parameters are provided in the PIDE, which were all fitted according to the same concept. This set of fitted LQ parameters was used in this work for RBE calculations and the validation of the LEM IV.

Most results of this study are shown exemplary for carbon ions for two reasons: first, a key application of the LEM I is in treatment planning for carbon ion therapy, and second, due to their relevance for therapy, experiments with carbon ions represent a major part of the PIDE. The purpose of this study is, however, a comprehensive model validation. Thus, the model accuracy is tested for several ion species in a broad energy range and the main results are presented for a variety of ions relevant for ion therapy and radiation protection.

## 2 MATERIALS AND METHODS

### 2.1 The local effect model

Carbon ions exhibit an increased RBE compared to photon and proton radiation, which depends on several parameters as LET, dose, or tissue type. Thus, for carbon ion treatment plan calculations an RBE model is required to accurately account for the variable RBE of carbon ions. For this purpose, the LEM was initially developed in the context of the carbon ion pilot project conducted at *GSI Helmholtzzentrum für Schwerionenforschung* (Darmstadt, Germany) from 1997–2008, where mainly patients with head‐and‐neck tumors were treated with carbon ion radiation.^1^, ^2^ Today, the model is applied at several cancer treatment facilities. Since its initial development, several model optimizations were performed leading to the current version of the LEM (LEM IV),^3^, ^4^ on which the investigations in this work are based. Although the general focus of the LEM application in therapy was on carbon ions, the recent model version can be applied to any other ion species as helium ions or oxygen ions, which also show an elevated RBE.

The LEM predicts the level of cell survival after ion radiation based on the cells’ response to photon radiation. The assumption is made that same local doses lead to same local effects within the cell nucleus—independent of the considered radiation quality. In the LEM version IV, the double‐strand‐break (DSB) distribution in the cell nucleus is calculated based on the local dose distribution. As extremely large local doses (> 100 Gy) may be present in the center of an ion track, additional DSBs may be formed due to interacting single‐strand‐breaks (SSBs), which is accounted for by the η‐factor.^16^ The radiation effect is then derived from the photon dose that is required to achieve the same damage complexity, as characterized by the spatial clustering of the DSBs with respect to mega base pair chromatin structures. This DSB clustering is more pronounced after ion irradiation as compared to photon irradiation, and according to the model, represents a key feature of ions leading to their increased effectiveness. Thus, for the prediction of cellular effects, such as clonogenic cell survival, the LEM requires the cells’ response to photon radiation as an input. This is provided in the form of the LQ parameters describing the photon survival curve, together with the specifications of the requested ion species (energy, LET). In this work, those four parameters were extracted directly from PIDE for each experiment. For all shown LEM IV simulations, the nuclear radius was set to 5 μm. A value of 5 μm serves as a reasonable first approximation and is typically used as default in LEM calculations. A specific consideration of the nucleus radius for each PIDE experiment separately would be desirable but is not feasible as the specific radius of a cell sample is in most cases not published by the authors and shows a size distribution itself. However, the size of the cell nucleus only influences the LEM simulations in the overkill regime and is chosen as an effective parameter to reflect the mean RBE of the distribution of cellular subpopulations.^17^ All other specific LEM IV parameters are applied as default as tabulated in.^17^ The detailed formalism of the LEM IV can be found in Equations (1)–(7) of ref 18; all simulations are performed with the clinically‐used single‐particle approximation.^2^

### 2.2 Quantification of model accuracy based on RBE

The most common model to describe cell survival curves mathematically is the LQ model^19^ with the cell survival *S*, dose *D*, and the linear–quadratic parameters α and β.

(1) $$-\ln\left(S\left(D\right)\right)=\alpha D+\beta D^{2}.$$

This form of the LQ model is used in this work to calculate RBE values based on the LQ parameters α and β, as given in the PIDE database. It is frequently discussed that cell survival curves re‐transition to a purely linear shape at large doses. As in the LEM framework the photon survival curve is extrapolated to very high doses, a modified version of the LQ model is used within the LEM. It improves the LQ model accuracy at higher doses by introducing a threshold dose *D*_t_. Above this dose, the cell survival curve is described by a purely linear shape, which better reproduces the experimentally found profile of cell survival curves at large doses.^20^, ^21^, ^22^ This results in the linear–quadratic–linear (LQL) model with the final slope $s_{\max}=\alpha +2\beta D_{t}$ and the DSB enhancement factor η:

(2) $$-\ln\left(S\left(D\right)\right)=\left\{\begin{array}{cc} \alpha D+\beta D^{2}, & D<D_{t}\\ s_{\max}\left(\eta \left(D\right)D-D_{t}\right)+\left(\alpha D_{t}+\beta D_{t}^{2}\right), & D\geq D_{t} \end{array}\right..$$

The threshold dose *D*_t_ is described with the empirically found formula^5^

(3) $$D_{t}=4\;\mathrm{Gy}+1.1\frac{\alpha_{\gamma }}{\beta_{\gamma }}.$$

The RBE is commonly used to quantify the effectiveness of a radiation quality for a specific endpoint, which is—in this study—cell survival. The RBE describes the ratio of doses of a reference radiation and the considered ion radiation, which lead to the same effect:

(4) $$\mathrm{RBE}={\left.\frac{D_{\mathrm{ref}}}{D_{i}}\right|}_{\mathrm{isoeffect}}.$$

As the RBE is dose dependent, the corresponding cell survival level needs to be specified. For instance, the RBE_10_ relates to the RBE at 10% cell survival. Moreover, the RBE_α_ refers to the RBE at zero dose limit *D*→0.

Each PIDE entry contains the LQ parameters of an irradiation experiment with a specific ion species as well as with a photon reference radiation. Thus, the RBE can be directly calculated for each PIDE experiment by transposing Equation (1) for the dose *D*, for both the ion and photon LQ parameters. The obtained doses *D*_i_ and *D*_ref_ can then be inserted into Equation (4) to calculate the RBE at a specific survival level.

For the validation of the LEM IV, for each experiment listed in the PIDE, the RBE is

1. directly calculated from photon and ion LQ parameters given in the PIDE;
2. predicted by the LEM IV based on the photon LQ parameters given in the PIDE.

For the LEM IV calculations, next to the photon LQ parameters α_γ_ and β_γ_, the desired ion species needs to be specified in the model input, as well as the ion's energy or LET. All other parameters were kept as default, as described in Friedrich et al.^17^ The application of the LQL model to PIDE data, which was originally fitted with the LQ model, is justifiable since the influence is small for RBE calculations. The RBE value is only affected by the choice of *D*_t_ if the considered effect level is connected to a dose larger than *D*_t_. For instance, the RBE_10_ is not affected for a vast majority of the included PIDE experiments since *D*_10 _< *D_t_* is true for the corresponding cell survival curves. At 1% cell survival (RBE_1_) still more than 400 of the 571 cell survival curves have not reached *D*_t_. Finally, the RBE values predicted by the LEM IV can be compared to the experimentally measured RBE values. The corresponding calculation scheme for the RBE and potential model deviations are shown in Figure 1.

**FIGURE 1:**
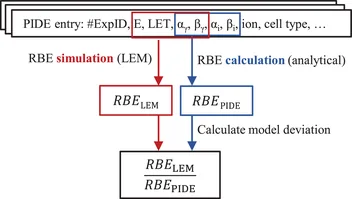
Schematic representation of the calculation and prediction of the RBE based on experimental data listed in the particle irradiation data ensemble (PIDE). From the measured (PIDE) and predicted (LEM) relative biological effectiveness (RBE) values, the model deviation can be determined.

### 2.3 RBE calculation and error estimation

As the accuracy of calculated RBE values directly depends on the precision of the LQ fits to the measured survival curves, the covariance matrices of the fits are used to determine RBE values and to estimate RBE uncertainties. Therefore, a Monte Carlo (MC) method is used as described in the following: potential pairs of α and β, which describe one specific survival curve, are found by random sampling from a bivariate normal distribution, which is defined according to the covariance matrix. In this work, 10 000 pairs of LQ parameters were sampled for each individual survival curve listed in the PIDE, reflecting the parameter space of α–β‐pairs in the range of their uncertainties, accounting for their correlation. For each parameter pair the dose at the considered cell survival level is calculated by Equation (1). As the sampling is done for the photon as well as for the ion survival curves, in the next step, dose pairs are matched randomly from the two pools of calculated doses. Finally, 10 000 RBE values are calculated by Equation (3) and the median of the obtained RBE distribution is taken as the final RBE value. Based on the RBE distribution, an RBE uncertainty is calculated as the half width of the central 68% quantile of the RBE distribution. This quantile is chosen, such that the uncertainty represents an equivalent to the standard deviation in a Gaussian normal distribution. Note that the median RBE values calculated with the MC method hardly vary from those determined by the simple RBE calculation with the single set of LQ parameters directly extracted from the PIDE. However, the application of the MC method allows providing reasonable error bars to the calculated RBE values as described in the previous paragraph. Calculating error bars using the conventional Gaussian error propagation is not applicable, as the uncertainty levels are in the order of magnitude of the corresponding quantities’ values and the RBE values follow a skew distribution. Exploring the uncertainty ranges by MC scanning methods gives thus a much more reliable error analysis.

While the uncertainty for the measured RBE values was obtained as described above, the attribution of an uncertainty to LEM predictions is more complex and needs to be determined considering the uncertainties of the model input parameters. However, since the observed uncertainties of the PIDE experiments reflect the typical precision of measurements in the corresponding LET regime they were also used to describe the accuracy of the corresponding LEM IV predictions.

### 2.4 Running averages to clarify trends of data

As the measurement data contained in the PIDE are subject to considerable spread, running averages through the data points are shown in several figures to guide the eye. Running averages represent a weighted averaging of data points, which lie in a specific data window. The running averages are used in this study in several figures, in which values are plotted as a function of LET. Thereby, a window size of one decade on the log scale was found to be a good compromise between smooth fit curves and a good representation of the data points by the fit. A weighting of all data within such an evaluation interval was performed using a Blackman window *w*_BM_(*x*, *x_i_*) and the absolute errors ∆*y_i_* to construct weights for each data point (*x_i_*, *y_i_*) in the considered window as

(5) $$w\left(x,\;x_{i}\right)=\;\frac{w_{\mathrm{BM}}\left(x,\;x_{i}\right)}{{\left(\Delta y_{i}\right)}^{2}}.$$

The standard error of the mean is used in order to enable an interpretation of the accuracy of the running average. In comparison to the standard deviation, it is proportional to $1/\surd N_{\mathrm{eff}}$ with *N*_eff_ as the effective number of measurement points included in the averaging process. Finally, the standard error of the mean SEM is equated by

(6) $$\mathrm{SEM}=\frac{\sigma }{\sqrt{N_{\mathrm{eff}}}}$$

with the standard deviation σ

(7) $$\sigma^{2}=\frac{\sum_{i}w\left(x,\;x_{i}\right){\left(y_{i}-\hat{y}\right)}^{2}}{\sum_{i}w\left(x,\;x_{i}\right)}$$

and the effective number of measurement points *N*_eff_

(8) $$N_{\mathrm{eff}}=\frac{{\left(\sum_{i}w\left(x,\;x_{i}\right)\right)}^{2}}{\sum_{i}w{\left(x,\;x_{i}\right)}^{2}}.$$

### 2.5 Criteria for data selection

The PIDE covers over 1100 cell survival experiments after irradiation. Each experiment is performed with a specific ion species as well as with a photon reference radiation under the same experimental conditions such as same cell line or cell cycle stage. The measured cell survival curves are described in the database by their corresponding LQ parameters. As the LQ parameters are obtained by different fitting procedures by different authors, next to the directly published LQ parameters, a set of LQ parameters is given, which was fitted by the authors of the database.^6^ This set of LQ parameters is chosen here for model validation purposes as the LQ parameters were determined directly from the raw data (measured dose‐survival points) and fitted according to the same concept for all cell survival curves.

Each experiment has a maximum dose and minimum cell survival level down to which the measurements were originally performed. To avoid extrapolations into dose regions not covered by experiments, the RBE calculations were restricted in a way that the RBE at a certain survival level was only calculated/predicted if the survival curves were measured down to the considered cell survival level. For instance, the RBE_1_ was only calculated if both, the survival curve for the ion as well as for the photon radiation, were measured down to a survival level of 1%.

The investigations are restricted to PIDE experiments, which were performed with monoenergetic ions only. Additionally, only experiments are considered for which the database authors provide standardized LQ fits, meaning that raw data of the measured survival curves were given in the original publications. Furthermore, to secure biological interpretation and to be compatible with the concept of the LEM, the choice of experiments was restricted to database entries with α_γ_ > 0, β_γ_ > 0, and α_i_ > 0. Negative values of β_i_ were allowed, even though, according to the LEM concept, no negative values of β_i_ will be predicted. A value of β < 0 points to the existence of at least two subpopulations of cells with different radiation sensitivities. The LEM, however, only considers cells with a uniform radiation response at once. One further experiment^23^ was excluded from this study as inconsistencies between the PIDE and published raw data were found. Finally, the experimental data used to determine the size of the megabase pair chromatin structures and the core radius of the ion track, which are used as fixed parameters in the LEM IV, were filtered out. The corresponding data are shown in Elsässer et al.^3^ (Figure 4) and comprise 39 more experiments to be excluded from this study. After the application of the above listed filter criteria, 571 experiments remain and their distribution concerning different ion species and other experimental characteristics is given in Table 1.

**TABLE 1:** Summary of the particle irradiation data ensemble (PIDE) data used in this study

Ion	H (^1^H+^2^H)	He (^3^He+^4^He)	^12^C	^20^Ne	^40^Ar	^56^Fe	Other	Sum
PIDE experiments	59 (45+14)	90 (27+63)	229	96	22	21	54	571
LET range, keV/μm	0.5–49.8	1.8–201	10.5–576	30–1245	81–2000	151.4–2106		
Energy range, MeV/u	0.465–179	0.275–199	1.69–440	1.74–415	4.64–680	13–1000		
Number of cell lines	18	25	49	9	8	9		
α_γ_, Gy^‐1^	0.01–0.96	0.05–2.16	0.02–1.17	0.05–0.51	0.06–0.36	0.05–0.51		
β_γ_, Gy^‐2^	0.001–0.093	0.005–0.145	0.001–0.157	0.007–0.167	0.007–0.046	0.013–0.056		
α_γ_/β_γ_, Gy	0.28–47.54	0.67–87.79	0.58–47.54	0.74–40.51	1.33–40.51	0.99–30.23		

Note: The number of experiment pairs of each ion species is listed together with other experimental characteristics. A minimum of 20 PIDE experiments is required for an ion to be included in the study. All ion species below this threshold are summarized under “Other” and are not part of this study due to lack of statistical power.

## 3 RESULTS

Figure 2 shows a scatter plot of the measured and the predicted RBE for the subset of 229 carbon ions. The results are shown for the RBE_α_ (a) at initial dose and the RBE_10_ (b) at 10% cell survival level. Furthermore, the data points for an LET ≤ 150 keV/μm are separated from data points obtained at higher LETs. This differentiation is performed, as an LET dependence of the model accuracy was found, pointing to an increased model accuracy in the so‐called overkill region. The RBE_α_ shows a large scatter up to an RBE of 25 connected to large error bars. Whereas the scatter of data is similar for the RBE_10_, it shows a smaller maximum RBE of about 6 as well as smaller error bars to the data points.

**FIGURE 2:**
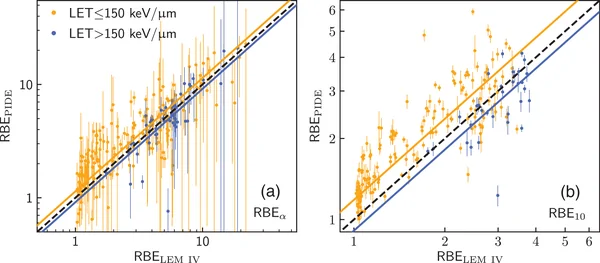
Scatter plot of the measured relative biological effectiveness (RBE) values (RBE_PIDE_) vs. the predicted RBE values (RBE_LEM IV_) for the RBE_α_ (a) and RBE_10_ (b). For both linear energy transfer (LET) subsets, a linear fit through the origin is plotted as a colored solid line together with a black dashed 1:1 correlation line representing optimal model predictions

Next to the data points, linear fits through the origin are shown for both LET ranges. In the fits, the error bars of both RBEs are accounted for, even if only the error bars of the measured RBE values are plotted. The error bars for the model predictions are not shown to keep the figures clear. As described in Section 2, for the LEM IV, the error bars are adapted from the corresponding PIDE experiments. In the case of RBE_α_, the slopes and standard errors of the fit curves are 1.14 ± 0.03 for an LET ≤ 150 keV/μm and 0.91 ± 0.05 for an LET > 150 keV/μm. Correspondingly, the slopes of the fit curves for the RBE_10_ are 1.18 ± 0.02 and 0.91 ± 0.03. Thus, the slope is in a similar order for both cell survival levels for the larger LET values. For lower LETs, however, the RBE underestimation is slightly larger for RBE_10_ than for RBE_α_. The coefficient of determination *R* ^2^ is 0.70 for RBE_α_ and 0.41 for RBE_10_.

In Figure 3, the RBE is plotted as a function of LET for carbon ion experiments. Measured RBE values as well as predicted values are presented together with a running average and its standard error to guide the eye. The general characteristics of an increasing RBE with LET can be clearly seen, as well as the drop in RBE for very high LETs due to the overkill effect. The model is able to reproduce the absolute values of RBE well, and also the peak position of the RBE maximum and the shape of the increase of RBE with LET in the low‐ to intermediate‐LET region. Similar to Figure 2, the RBE_α_ shows a large scatter. For both cell survival levels, the predictions of the RBE lie on average slightly below the measured RBE values for LETs < 150 keV/μm (b). For larger LETs, in the overkill region, the model shows a tendency to overestimate the RBE.

**FIGURE 3:**
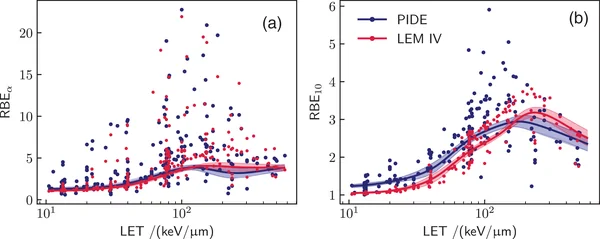
Relative biological effectiveness (RBE) as a function of linear energy transfer (LET) for carbon ion experiments listed in the particle irradiation data ensemble (PIDE) as well as predicted by the local effect model IV (LEM IV). The characteristics are shown at two different cell survival levels together with the corresponding running averages (solid lines) and their standard errors as error bands. The error bars of individual data points are not shown for the sake of visibility but are included in the calculation of the running averages

To investigate the observed characteristics in more detail, the RBE deviations of the model predictions are calculated according to the concept shown in Figure 1. In Figure 4a, the deviation of the LEM IV for carbon ions is shown for the RBE_10_ together with its running average and error bars. In Figure 4b, the deviations are shown for several RBEs at different cell survival levels, again for carbon ions. For all cell survival levels the same trends are found. The RBE is underestimated at an LET < 100–200 keV/μm for about −20%, which is, however, in the range of the size of the error bars. With increasing LET values the accuracy of the predicted RBE rises and at LETs > 200 keV/μm no clear model deviation is observed within the limits of the statistical power. Figure 4c shows the model deviations for RBE_10_ for several ions ranging from protons to iron ions. All considered ion species follow a similar trend as carbon ions and show an underestimation of RBE at lower LET values; the degree of RBE underestimation rises with the ions’ atomic number. For higher LETs, the characteristics differ between different ion species. Whereas the predictions in this LET region show high accuracy for carbon ions and heavier ions, for lighter ions as proton and helium ions the model tends to overestimate the RBE. In order to facilitate the comparison of different models, it is tempting to condense all information about deviations in a single number like, for example, *χ* ^2^ that should uniquely characterize the model accuracy. However, even if such a number seems to be illustrative, it is related to substantial pitfalls, as demonstrated in Figure 4d, where the average model deviation and the corresponding range of deviations are shown for each particle species. These average deviations were calculated as the mean of log_10_(RBE_10,LEM_/RBE_10,PIDE_), where the running averages were used to calculate the mean instead of specific data points. Furthermore, the averaging was performed over a logarithmic LET scale. The logarithmic vertical scale was chosen since in this representation an over‐ and underestimation of RBE by the same factor corresponds to the same visible length. Since with this method an equal over‐ and underestimation may cancel each other out, error bars are shown additionally, which allow to assess the range of deviations around the mean deviations. The error bars are calculated as the mean of the absolute (unsigned) deviation of the running averages with respect to the previously calculated average model deviation. The average model deviations are summarized in Table 2 for each ion species and cell survival level. While we show these values mainly for illustrative purposes, we also highly encourage the reader to handle such values with caution for several reasons:

1. They do not reflect any systematic trends within the parameter range, and thus models with similar mean values and range of deviations might substantially differ with respect to their systematic dependencies of deviations on the relevant model parameters.
2. In particular, they do not reflect, for example, the direction of systematic dependencies of deviations on the LET. For instance, if two RBE models both show LET‐dependent deviations, where one model underestimates the RBE at low‐LET values and overestimates it at high‐LET values, and the second shows the opposite trend, this cannot be discriminated based on these mean numbers. As a result, the corresponding model assessment with respect to clinical applications might be entirely different, depending on whether one is dealing with low‐LET values and thus preferentially with effects in the surrounding normal tissue or with high‐LET values and thus preferentially with effects in the target volume.
3. The use of running averages requires sufficient experimental data that are almost homogenously spread throughout the relevant parameter range. Although in principle mean deviations obtained from integration over running averages can be substituted by summing up deviations over the corresponding individual data points, this can lead to substantial bias if data points are not evenly distributed over the considered parameter range (in this case LET). Additionally, too few data may lead to the problem that stochastic fluctuations around the mean value cannot be distinguished from systematic trends over the relevant LET range.*

**FIGURE 4:**
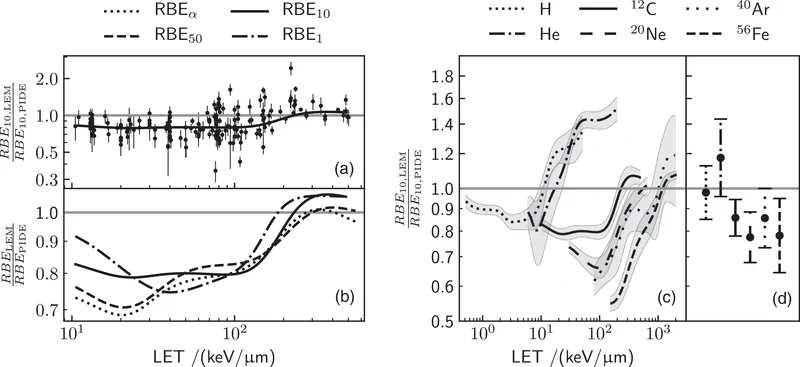
Relative model deviations of relative biological effectiveness (RBE) for carbon ions as predicted by the local effect model IV (LEM IV) compared to measurements listed in the particle irradiation data ensemble (PIDE) for several cell survival levels (a,b). Similar trends of an increasing model accuracy with linear energy transfer (LET) are also found for other ion species (c). The lines represent running averages to the data points. The averages of the running averages in (c) are graphically represented in (d) together with error bars. These error bars visualize the degree of fluctuation of the model deviations

**TABLE 2:** Average model deviation of relative biological effectiveness (RBE) for several cell survival levels and ion species evaluated over the entire linear energy transfer (LET) range covered by experimental data in the particle irradiation data ensemble (PIDE)

Ion	H (^1^H+^2^H)	He (^3^He+^4^He)	^12^C	^20^Ne	^40^Ar	^56^Fe
RBE_α_	$0.93_{-0.18}^{+0.22}$	$1.14_{-0.16}^{+0.19}$	$0.82_{-0.09}^{+0.10}$	$0.83_{-0.10}^{+0.12}$	$0.83_{-0.13}^{+0.15}$	$0.61_{-0.09}^{+0.11}$
RBE_50_	$0.96_{-0.13}^{+0.15}$	$1.10_{-0.16}^{+0.18}$	$0.83_{-0.09}^{+0.09}$	$0.82_{-0.11}^{+0.12}$	$0.81_{-0.10}^{+0.11}$	$0.68_{-0.11}^{+0.13}$
RBE_10_	$0.98_{-0.13}^{+0.15}$	$1.17_{-0.22}^{+0.27}$	$0.86_{-0.08}^{+0.09}$	$0.77_{-0.10}^{+0.11}$	$0.86_{-0.13}^{+0.15}$	$0.78_{-0.14}^{+0.17}$
RBE_1_	$1.17_{-0.05}^{+0.06}$	$1.11_{-0.13}^{+0.15}$	$0.87_{-0.10}^{+0.11}$	$0.73_{-0.12}^{+0.14}$	$0.78_{-0.14}^{+0.16}$	$0.82_{-0.26}^{+0.37}$

Note: Since the error bars are asymmetric on a non‐logarithmic scale, they are provided in the index/exponent notation.

In Figure 5, the LEM IV is tested concerning its ability to reproduce the characteristic dependence of RBE on the inverse α_γ_/β_γ_ ratio of the photon survival curve. The RBE_α_, the RBE_50_ and the RBE_1_ are shown for PIDE experiments for carbon ions in the LET range of 70 ≤ LET < 80 keV/μm. For each experiment, the RBE was directly calculated as well as predicted by the LEM IV. Linear fits were calculated including RBE uncertainties, and the corresponding fit values are given in Table 3. The increase of RBE with increasing β_γ_/α_γ_ ratio can be seen for the RBE_α_, whereas no RBE dependence on β_γ_/α_γ_ is found for the other RBE levels. This behavior confirms findings previously shown in Friedrich et al.^5^ for a smaller subset of experiments. The LEM IV is able to well reproduce the experimentally found characteristics of the RBE dependence on β_γ_/α_γ_. The data presented in Table 3 indicate that although for RBE_50_ and RBE_1_ no significant deviation is observed between the two fit curves, a deviation is seen for RBE_α_. The larger offset for the fit curve to the PIDE data of RBE_α_ might be due to the single data point (PIDE ID no. 978, top left in the figure, with β_γ_/α_γ _= 0.027 Gy^–1^ and α_γ_ = 0.268 Gy^–1^, β_γ_ = 0.007 Gy^–2^, α_i_ = 1.5879 Gy^–1^, β_i_ = −0.029 Gy^–2^). Note, however, that the presented linear fit is an approximation and is primarily shown to guide the eye. The real characteristic shape of RBE as a function of β_γ_/α_γ_ might be more complex as already observed in Friedrich et al.^5^

**FIGURE 5:**
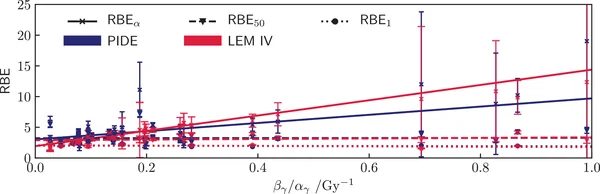
Relative biological effectiveness (RBE) as a function of the β_γ_/α_γ_ ratio for carbon ions at an linear energy transfer (LET) ≥ 70 and < 80 keV/μm. 27 of the 229 experiments conducted with carbon ions are included in this LET window; due to data filtering as described in the Section 2, 27(24), 26(23), and 7(7) RBE values remain for the RBE_α_, RBE_50_, and RBE_1_, respectively. The values in parentheses denote the number of different cell lines included. Measured values for the RBE_α_, RBE_50_, and RBE_1_ are shown as blue crosses, triangles, and dots, respectively. For each experiment, the RBE is also predicted by the local effect model IV (LEM IV) (red data points). For both, measurement and model prediction, linear fits through the data points are presented. The *x*‐axis is limited to a value of 1, therefore, one pair of data points with β_γ_/α_γ_ = 1.72 Gy is not shown but included in the data fitting process

**TABLE 3:** Fit values with standard deviations for the function *f*(β_γ_/α_γ_) = *a*(β_γ_/α_γ_)+*b* shown in Figure 5 for the relative biological effectiveness (RBE) as a function of β_γ_/α_γ_ for several cell survival levels

	PIDE			LEM IV		
	RBE_α_	RBE_50_	RBE_1_	RBE_α_	RBE_50_	RBE_1_
*a*	6.68 ± 2.30	0.04 ± 0.52	−0.12 ± 0.27	12.45 ± 0.76	0.46 ± 0.30	−0.32 ± 0.31
*b*	3.02 ± 0.32	3.23 ± 0.21	2.01 ± 0.14	1.93 ± 0.11	2.90 ± 0.13	2.07 ± 0.15

In Figure 6, the ratios α_i_/α_γ_ and β_i_/β_γ_ are plotted as a function of the LET to elucidate the LET dependence of the deviations of the predicted ion LQ parameters. The results are shown for carbon ions as well as for hydrogen and iron ions, which represent the lightest and heaviest ion species considered in this study, respectively. In accordance to Figure 2, an underestimation of α_i_ and β_i_ can be seen for carbon ions at LETs < 150 keV/μm, directly leading to an underestimation of RBE. At very high LETs a small overestimation of β_i_ is found, which is a consequence of the current implementation of the approximation method of the LEM, more specifically, the manner how the η‐factor is included in the determination of the LQ parameter β_i._ ^2^, ^16^ However, in order to be able to make a statistically solid statement of the degree of overestimation, further simulation studies for heavier ions need to be performed. The same translation of deviations in the LQ parameters into RBE was also found for protons and iron ions. An underestimation of α_i_ and β_i_ directly results in an underestimation of RBE and vice versa (compare to Figure 4c). For all three ion species, a few experiments are found to show negative values of β_i_/β_γ_, which points to the existence of multiple subpopulations of cells with different radiosensitivities. As the LEM only considers one cell type with a specific radiosensitivity per simulation (defined by the LQ parameters), no negative values of β_i_ are predicted. Note that PIDE experiments with β_γ_ ≤ 0 were excluded from the study.†

**FIGURE 6:**
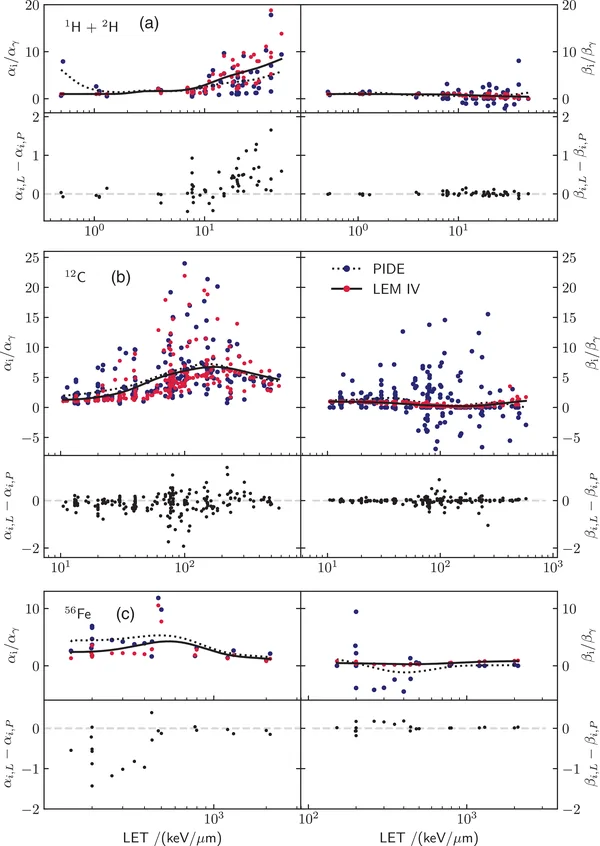
Top panels: data points with running averages for the ratios α_i_/α_γ_ ( = RBE_α_) and β_i_/β_γ_ as a function of linear energy transfer (LET) for the predicted and measured values of protons and deuterons (a), carbon ions (b), and iron ions (c). Bottom panels: corresponding absolute model deviations. P: PIDE, L: LEM; the gray dashed line indicates a horizontal line at an absolute model deviation of 0

## 4 DISCUSSION

The investigations demonstrate that the LEM IV enables a reasonable RBE prediction for various experimental scenarios. The model was tested for numerous ion species relevant in ion radiotherapy and radiation protection, for cells of different radiosensitivity, and in general for ions in a broad energy range. Furthermore, the LEM IV is capable to predict the dependence of RBE_α_ on the inverse α_γ_/β_γ_ ratio of the photon survival curve as visible from the slope parameters in Table 3. The non‐existent or very small dependence of the RBE_50_ or RBE_1_ on the inverse α_γ_/β_γ_ ratio is also reproduced by the LEM IV. As the applied dose as well as the cells’ α_γ_/β_γ_ ratio are typical parameters for treatment planning, this feature is important for the benchmarking of any RBE model. The presented work comprises a systematic model test with in vitro cell survival data. Note that when moving to more realistic clinical scenarios other parameters as genomic variabilities from tumor to tumor or even within the same tumor would need to be considered. However, assuming that genomic variability translates into corresponding variations in photon radiosensitivity, the LEM can be applied in these situations as well, as soon as the photon sensitivity can be characterized by the corresponding LQ‐parameters. The presented investigation of model deviations revealed certain systematics. For instance, for carbon ions at low‐LET values, a tendency of RBE underestimation was found, as shown in Figures 2 and 3. This characteristic of LEM IV was observed earlier for V79 Chinese hamster cells in vitro^4^ as well as for the tolerance of rat spinal cord in vivo^12^ by comparison to single measurement datasets. Furthermore, underestimations of RBE for carbon ions in the entrance channel were recently reported by Mein et al.^13^ for four different tumor cell lines. The results of this work support these findings based on model comparison to a large dataset. Note that in contrast to that, for LEM I an overestimation of RBE is observed at small LET values.^24^ For other ion species than carbon ions, the LEM IV deviation of RBE as a function of LET was found to follow a similar shape as for carbon ions of an increased model accuracy with increasing LET. Protons and helium ions additionally show an overestimation of RBE at large LETs.

Furthermore, this study demonstrates the value and importance of large experiment databases as the PIDE for general RBE/survival model testing. Next to the application in this work, databases as the PIDE are frequently used to fit or validate high‐LET models. ^25^, ^26^, ^27^ However, whereas these works either filtered the PIDE data for specific ion species or cell lines, this work comprises the first study in which a high‐LET model is systematically tested for multiple ion species and energies by comparison to more than 570 in vitro cell survival experiments including all cell lines available in the PIDE. The key role of applying databases in that regard is that they contain independent experiments. This independence of data points allows detecting systematic uncertainties in model predictions, even if they are smaller than the average experimental uncertainty of individual datasets. Analogous to the model assessment of LEM IV presented in this work, the database can also be applied in a similar manner to test other RBE or cell survival models. This enables a comprehensive comparison of several biophysical models with each other, for instance concerning their accuracy in different LET ranges. For such comparisons involving PIDE, a goodness‐of‐fit measure is needed to quantify the proximity between model predictions and the experimental data. In order to compare different models, it is important that such a measure accounts for the number of free parameters (= degrees of freedom), as, e.g., considered in a calculation of reduced *χ* ^2^ values. Besides those parameters, which specify the experiment to be simulated, the idea of the LEM is to make model predictions based on a minimum possible number of degrees of freedom. Despite LEM IV involves some general parameters, it does not rely on experiment‐specific free parameters, which would be adapted to individual experiments or groups of experiments.^17^ Here, the nomenclature of Friedrich et al.^17^ is used defining two groups of LEM input parameters: “specific parameters, which specify the situation to be modeled, and general parameters, which have been obtained by measurements, fitted to reference data or estimated by theoretic considerations.”

Generally, a large number of parameters usually leads to enhanced uncertainties in any of these parameters, which often complicate an interpretation of their values. In contrast to that, the LEM formalism enables an understanding of relevant mechanistic physical and biological interactions of radiation with biological targets. Although conceptually different in several aspects, the microdosimetric kinetic model^28^, ^29^, ^30^, ^31^ also shares some similarities with the LEM, for example, with respect to the relevance of the micrometer scale, which according to the LEM can be considered as one important scale to explain the increased effectiveness of high‐LET radiation. A direct comparison of the model accuracy of both models will thus be of particular interest and corresponding activities are ongoing. Other biophysical models exist that use several fit parameters or gauging functions optimized specifically for one situation, for example, for a specific radiation quality or a defined survival level.^32^, ^33^, ^34^ Thus, strictly speaking, they can be rather understood as fit models instead of truly predictive models, as they already need high‐LET data to predict effects after exposure to high‐LET radiation. However, such models nevertheless are of use, as from the dependence of the free parameters on the radiobiological settings one may challenge their biological or physical meaning. Finally, an extreme example of parameter rich models would be a simple polynomial fit of sufficient high order to available RBE data. In a similar manner, machine learning models can be trained with the PIDE data and used for RBE predictions. However, first tests carried out by the authors revealed that in both cases extrapolation into physical regions where no measurement data is available are difficult and may give diverging results, as the approaches are not based on the simulation of physical nor biological features and interactions. For the same reason, the involved parameters cannot be associated with a proper radiobiological meaning.

The systematic quantification of RBE predictions revealed a systematic underestimation of RBE at lower LETs. Furthermore, the model's accuracy was found for the RBE_50_ and RBE_1_ to be independent of the α_γ_/β_γ_ ratio (Figure 5), which indicates that the model deviation is probably not of biological nature. Thus, the simulation of physical properties might be the origin of the observed deviations. In many experiments performed with passive beam delivery, large material blocks are used in experiments to reduce the beam energy for measurements. Thus, especially target fragments are produced, which are typically of lower energy and are, therefore, highly effective. Thus, cell survival measurements could lead to artificially increased RBE values. However, due to their low energy, target fragments also exhibit very small ranges and thus, probably would hardly reach the biological sample. In the current implementation, the LEM IV does not include any projectile or target fragmentation along the ion's track through the cell nucleus. Second, in the LEM, the assumption is made that same local photon doses and ion doses lead to the same effect. Thus, the underlying secondary electron spectra induced by photons or ions are not specifically considered in the RBE determination. However, the majority of DNA damages are induced by electrons. And since the secondary electron spectra of ions and photons differ considerably and the RBE for DSB induction of electrons depends on electron energy, also different effects are expected for different primary radiation species—even at the same local dose. In a new model approach, the mean RBE of the present secondary electron spectra will be determined in dependence of the radial distance to the ion track. This electron RBE as a function of the radial distance can then be used to weight the physical dose profile before continuing with the typical LEM IV calculation procedure. Thus, the same local doses of photons and ions are not considered to necessarily lead to the same effect any more as the effectiveness depends on the underlying secondary electron spectra. This new approach is implemented in a future version of the LEM, which will be content of a forthcoming paper.

## 5 CONCLUSIONS

In this study, the RBE‐predictive high‐LET model LEM IV is systematically tested by comparing simulation results to measurement data given in the PIDE. The ability to correctly predict the RBE is used as a general measure of the model's precision.

The investigations in this work demonstrate the universal applicability of the LEM IV concept for the simulation of various experimental scenarios, covering several ion species in a broad energy range. As shown in various publications before, the LEM IV is generally able to reproduce the characteristic shape of RBE as a function of LET.^3^, ^4^, ^7^, ^8^, ^9^, ^10^, ^11^ Furthermore, due to the detailed quantitative comparison of simulation results to PIDE measurement data, previously reported model limitations could be confirmed. For lower LET values, the LEM IV tends to underestimate the RBE and the model accuracy decreases with increasing atomic number of the considered ion. At higher LET values, the model results are in good agreement with measurement data for carbon ions and heavier ions. However, for lighter ions an overestimation of RBE was found. As a result, the strengths and weaknesses of the LEM IV were determined enabling the development of further model optimizations with the aim to improve RBE predictions in the relevant LET ranges. Such optimizations are currently under development.

## CONFLICTS OF INTEREST

M. Scholz holds a patent on the LEMIV under EP 2 670 484. All other authors declare no conflict of interest.

## Supporting information

Supporting Information

Supporting Information

## Data Availability

The data shown in the figures of this study are available in the supporting information of this article. Furthermore, information about excluded data (due to earlier usage as model training data) is provided.

## Footnotes

* The error bars of the RBE deviations, which are used in the calculation process of the running averages were calculated by Gaussian error propagation.

† In the panel for ^12^C ions, the *y*‐axis was cut off at a value of β_i_/β_γ_ = −9, to ensure visibility of the differences in the fit curves. There are five more PIDE data points for β_i_/β_γ_, which lie in the range of β_i_/β_γ_ = −9 and β_i_/β_γ_ = −24 and are thus not shown in the plot.
