# Supplementary material for: Comprehensive comparison of local effect model IV predictions with the particle irradiation data ensemble
Source: Med Phys. 2021 Dec 1;49(1):714–26. doi: 10.1002/mp.15343 (PMC13655482; doi:10.1002/mp.15343)
Supplement: Supplementary file 1 [file MP-49-714-s001.docx]

After application of the filter criteria to the PIDE 3.2 as described in the publication, 610 experiments remain.

Out of these, 39 more experiments were excluded as these were previously used to fit the LEM IV parameter l_DSB_ (See Elsässer T, Weyrather WK, Friedrich T, Durante M, Iancu G, Krämer M, et al. *Quantification of the relative biological effectiveness for ion beam radiotherapy: Direct experimental comparison of proton and carbon ion beams and a novel approach for treatment planning.* Int. J. Radiat. Oncol. Biol. Phys. 2010; 78:1177–83).

The corresponding 39 excluded experiment IDs in the PIDE 3.2 are:

- 62, 63, 64 (^1^H, folkard96)
- 219, 220, 221, 222, 223, 224, 225, 226, 227, 228, 229, 230 (^3^He, furusawa00)
- 233, 234, 235, 236, 237, 238, 239, 240, 241, 243, 244, 245, 246, 247, 251 (^12^C furusawa00)
- 416, 417, 418, 419, 420, 421 (^1^H, belli98)
- 438 (^1^H, perris86)
- 442, 443 (^4^He, cox77)
